# Use of intervention mapping to adapt a health behavior change intervention for endometrial cancer survivors: the shape-up following cancer treatment program

**DOI:** 10.1186/s12889-018-5329-5

**Published:** 2018-03-27

**Authors:** Dimitrios A. Koutoukidis, Sonia Lopes, Lou Atkins, Helen Croker, M. Tish Knobf, Anne Lanceley, Rebecca J. Beeken

**Affiliations:** 1 Institute for Women’s Health, University College London, London, UK; 2 Nuffield Department of Primary Care Health Sciences, University of Oxford, Oxford, UK; 3 Department of Behavioural Science and Health, University College London, London, UK; 4 Centre for Behaviour Change, University College London, London, UK; 5 School of Nursing, Yale University, New Haven, CT USA; 6 Leeds Institute of Health Sciences, University of Leeds, Leeds, UK

**Keywords:** Healthy eating, Physical activity, Behavior change, Endometrial cancer, Survivorship, Intervention mapping

## Abstract

**Background:**

About 80% of endometrial cancer survivors (ECS) are overweight or obese and have sedentary behaviors. Lifestyle behavior interventions are promising for improving dietary and physical activity behaviors, but the constructs associated with their effectiveness are often inadequately reported. The aim of this study was to systematically adapt an evidence-based behavior change program to improve healthy lifestyle behaviors in ECS.

**Methods:**

Following a review of the literature, focus groups and interviews were conducted with ECS (*n* = 16). An intervention mapping protocol was used for the program adaptation, which consisted of six steps: a needs assessment, formulation of matrices of change objectives, selection of theoretical methods and practical applications, program production, adoption and implementation planning, and evaluation planning. Social Cognitive Theory and Control Theory guided the adaptation of the intervention.

**Results:**

The process consisted of eight 90-min group sessions focusing on shaping outcome expectations, knowledge, self-efficacy, and goals about healthy eating and physical activity. The adapted performance objectives included establishment of regular eating, balanced diet, and portion sizes, reduction in sedentary behaviors, increase in lifestyle and organized activities, formulation of a discrepancy-reducing feedback loop for all above behaviors, and trigger management. Information on managing fatigue and bowel issues unique to ECS were added.

**Conclusions:**

Systematic intervention mapping provided a framework to design a cancer survivor-centered lifestyle intervention. ECS welcomed the intervention and provided essential feedback for its adaptation. The program has been evaluated through a randomized controlled trial.

**Electronic supplementary material:**

The online version of this article (10.1186/s12889-018-5329-5) contains supplementary material, which is available to authorized users.

## Background

The high prevalence of obesity and suboptimal lifestyle behaviors render the increasing population of endometrial cancer survivors a high-risk group for both mortality and morbidity [1, 2]. Obesity is negatively associated with health-related quality of life (HRQoL) in this population [3] and may be associated with lower overall survival in the long term [4]. Only a few survivors seem to spontaneously improve their eating and activity patterns post-diagnosis [5], although relevant guidelines exist [6] and a cancer diagnosis has been described as a “teachable moment” [7]. Qualitative work with endometrial cancer survivors suggests they try to make lifestyle changes post-diagnosis, but experience cancer-specific barriers, such as fatigue and bowel issues, and feel there is a lack of guidance. Furthermore, patients report a desire for in person advice post-treatment [8].

There is, therefore, a need for effective lifestyle behavior change interventions that can help this high-risk group to adopt and sustain health-protective behaviors [9]. However, the limited available interventions in endometrial cancer survivors are resource intensive, and non-UK based [9, 10] which may render their dissemination within the UK health service challenging. A promising approach may be to take an existing evidence-based intervention with proven effectiveness in the general population in the UK, and adapt it for endometrial cancer survivors. Such an approach could increase the potential for effective adoption, implementation, and sustainability, should the adapted version prove successful.

Applying an intervention to a different population requires adaptation. Some of the evaluated lifestyle programs in cancer survivors have used adapted versions of evidence-based programs. For example, the LEAN (Lifestyle, Exercise, and Nutrition) study used an adaptation of the Diabetes Prevention Program [11]. However, most lifestyle interventions in cancer survivors inadequately report their theoretical constructs and behavioral change techniques (BCTs) [12]. Adaptation can be done under a systematic framework based on Intervention Mapping; IM adapt. Intervention mapping (IM) is a six-step framework for developing theory-based and evidence-based health promotion programs [13]. The IM adapt approach ensures the needs and preferences of the new population are considered, the intervention is accurately described, and its effective core components are retained [14].

An existing evidence-based weight management behavior change program is currently used across the UK in health and community settings. Developed by Weight Concern, “Shape-Up” is an eight-week, group-based program comprised of a self-help manual for participants [15] and a manual for facilitators [16]. It focuses on establishing a healthy diet and increasing physical activity to achieve modest weight loss. A version of this program has been favorably evaluated in terms of acceptability, physical, and psychological outcomes over one-year follow-up [17]. It is based on Social Cognitive Theory (SCT) [18] and Control Theory (CT) [19], in line with previous research that indicates the potential effectiveness of the two theories [12, 20]. Thus, “Shape-Up” might be a unique intervention that could be delivered outside the research setting. The aim of this study was to systematically adapt “Shape-Up” for endometrial cancer survivors using the IM adapt approach.

## Methods

Shape was adapted using the six-steps within the IM Adapt approach (Table 1). This enabled us to systematically adapt the intervention through comparing the logic model of change for the original program with the needs of the new population [14]. Details of each step are outlined below.

**Table 1:** Overview of the 6-step IM Adapt process

Steps	Description
Step 1	Needs assessment, organizational capacity, and logic models
1a	Describe organizational capacities and goals
1b	Conduct a needs assessment to describe the health/behavior problems and develop a logic model of the problem
1c	Develop a logic model of change
1d	Write program goals
Step 2	Search for evidence-based interventions (EBIs)
2a	Search for EBIs to address the health problem/ risk behavior/ environmental factor
2b	Judge basic fit to health problem, behavior, priority population, and organizational capacity
Step 3	Assessment of detailed fit and planning of adaptations
3a	Judge behavioral and environmental fit and list adaptations
3b	Judge determinants and change methods fit and list adaptations
3c	Judge delivery, design, and cultural fit and list adaptations
3d	Judge implementation fit and list adaptations
3e	Identify essential elements of the selected intervention and how to retain them
Step 4	Adaptation of materials and activities
4a	Prepare design documents for adaptation
4b	Pre-test adapted materials
4c	Produce final adaptations
Step 5	Planning of implementation
5a	Identify facilitators, facilitation behaviors, and outcomes
5b	Develop facilitation scope, sequence, and instructions
5c	Plan activities to motivate and train for facilitation
5d	Plan logistics including budget, staffing, and materials
Step 6	Planning of evaluation
6a	Write evaluation questions
6b	Choose indicators and measures
6c	Choose the evaluation design
6d	Planning data collection, analysis, and reporting

### Step 1 Needs assessment, organizational capacity, and logic models

Following an assessment of organizational capacity and goals (1a), the adaptation process included a needs assessment examining the health and HRQoL issues in endometrial cancer survivors and their association with lifestyle behaviors (1b). These were conducted through a literature review and a qualitative study that took place in London, UK. All constructs of SCT (i.e. outcome expectations, knowledge, self-efficacy, and goals) were included in the model as determinants of healthy eating and physical activity, because a comprehensive use of theory is associated with intervention effectiveness [21].

Subsequently, a logic model of the problem linked the behaviors with health problems and HRQoL. This formed the basis for the development of the logic model of change that illustrates how the theoretical change methods influence the behavioral determinants that, in turn, affect the behaviors and, subsequently, health and HRQoL (1c). The performance objectives represent observable subsets of the targeted behaviors and the change objectives describe what participants must learn or change to meet or maintain the performance objective. Given the organizational capacity, the models were only focused on shaping the behavioral aspects of the problem. Lastly, the detailed program goal was established (1d).

### Step 2 Search for evidence-based interventions (EBIs)

While the “Shape-Up” program was the initial choice, a retrospective search for other evidence-based interventions (2a) was run to confirm that no better option existed (Additional file 1). Furthermore, the basic fit of the chosen program and its acceptability was assessed by providing endometrial cancer survivors who participated in a previous qualitative study [8] with the “Shape-Up” booklet and prompting them for their initial thoughts (2b).

### Step 3 Assessment of detailed fit and planning of adaptations

This step examined the fit between the logic model of behavior change for endometrial cancer survivors (3a) and the selected program and evaluated the fit of the determinants and the theoretical methods of the original program for the new setting (3b). Additionally, it assessed the design, delivery and cultural fit (3c), considered its implementation fit (3d), and derived the essential elements of the original program that should be retained (3e).

The behavioral change techniques used in the original intervention were retrospectively coded using the behavior change technique taxonomy v1 (BCTTv1) [22]. The assessment also included defining the components of the logic model of change and formulation of the matrices of change objectives for the original program. Each matrix of change objectives links each behavior with the behavioral determinants, the performance objectives, the change objectives, the practical application of the change objectives, the BCTs, and the theoretical methods. This was an essential step towards improving the reporting of the intervention [23].

The content update was based on (i) current guidelines for cancer survivors [6], and (ii) the current evidence and resources on healthy eating and physical activity. A thematic analysis of qualitative interviews providing feedback on “Shape-Up” informed the delivery, design, and cultural fit [24, 25]. The methods for this analysis are described in Additional file 1. The implementation fit was judged based on the delivery method and the service evaluation of the original program. Furthermore, the program was presented at the National Cancer Research Institute (NCRI) Gynecological Cancer Cervix/Vulva and Endometrium Workshop in 2014. It was also discussed with a registered dietitian working with cancer survivors.

Given the lack of qualitative research and formal evaluation of change objectives in the original program, identification of the essential elements was based on both feedback from the evaluation of “Shape-Up” in the community [26] and consideration of the evidence from systematic reviews and guidelines on behavior change [20, 27–29].

### Step 4 Adaptation of materials and activities

The fourth step included the preparation of the new design documents (4a), pretesting of the adapted materials (4b), and production of the final versions (4c). The drafted documents were reviewed in an iterative process ensuring they appropriately resembled the matrices of change objectives. Time constraints prohibited the originally planned pretesting of the materials.

### Step 5 Planning of implementation

In the fifth step, facilitators, facilitation behaviors, and outcomes were specified (5a). The scope, sequence, and instructions for facilitation were developed (5b). The matrices of change objectives for facilitators were retrospectively created based on the original facilitator’s manual and were matched to SCT and CT constructs. These followed the rationale of the matrices in Step 3. A modified version of the original facilitator’s manual was created to reflect the content changes in the adapted version. These were followed by planning training and motivation activities for implementers (5c), and planning of logistics (5d).

### Step 6 Planning of evaluation

The final step consisted of the development of the evaluation questions (6a), selection of indicators and measures (6b), selection of the evaluation design (6c), and planning of the collection, analysis, and data reporting (6d).

## Results

### Step 1 Needs assessment, organizational capacity, and logic models

#### 1a. Organizational capacity

University College London (UCL) initiated the program planning focused on developing a healthy eating and physical activity intervention for endometrial cancer survivors to improve their HRQoL. To do so, it collaborated with the charity Weight Concern and the final team involved all authors.

#### 1b. Needs assessment and logic model of the problem

**Fig. 1:**
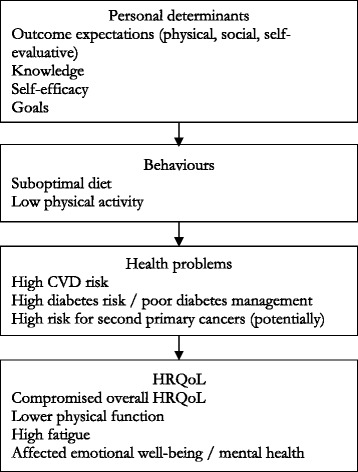
Logic model of the problem

The needs assessment highlighted the high disease burden and need for an intervention [3, 8]. The logic model of the problem was constructed (Fig. 1), and SCT and CT guided the adaptation of the intervention in line with the theoretical basis of the original “Shape-Up” and the literature. A meta-regression of effective BCTs identified self-monitoring paired with at least one additional technique from CT to be significantly more effective compared to other interventions [20] and a recent systematic review of behavior change interventions in cancer survivors based on SCT indicated their effectiveness for improving physical activity immediately post-intervention and for promoting dietary change [12].

#### 1c. Logic model of change

**Fig 2:**
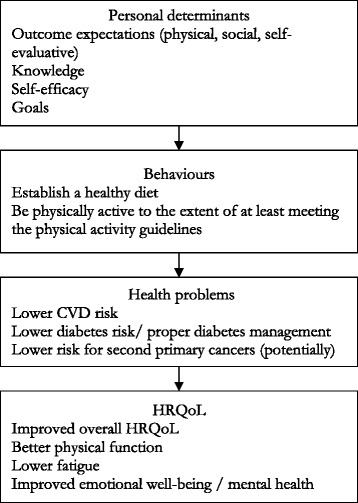
Logic model of change in this population

In this step, the logic models of changes for both the original and the adapted programs were developed. The main difference between the two was the removal of weight loss in the latter model. The adapted model is presented in Fig. 2. It postulated that outcome expectations, knowledge, self-efficacy, and goals (personal determinants) would help individuals to establish a healthy diet and increase physical activity to the extent of at least meeting the physical activity guidelines (behaviors). This would, in turn, lower their disease burden (health problems) and improve their HRQoL (HRQoL outcomes).

#### 1d. Program goals

Given the above, the goal of the program was to produce small clinically significant improvements in the overall HRQoL of participants in the first 6 months of program implementation [30].

### Step 2 Search for evidence-based interventions (EBIs)

#### 2a. Search for EBIs

The retrospective search of the Internet, PubMed, and databases for effective evidence-based interventions did not return any intervention that would be a good fit for the current population. As the correlates of behavior appear to be similar for endometrial cancer survivors and the general population [8], the search confirmed that “Shape-Up” could be an ideal candidate program for evaluation.

#### 2b. Judge basic fit

The targeted behaviors (establishing a healthy diet and increasing physical activity to the extent of at least meeting the physical activity guidelines) were the same for the general population and endometrial cancer survivors. Additionally, the organizational capacity could support the program, as the intervention did not require specific equipment or space but could be run in local places, such as community centers. Finally, its acceptability based on the initial thoughts of the participants was high and this was further reinforced through the telephone interviews detailed below.

### Step 3 Assess (detailed) fit and plan adaptations

Details on specific changes on the manual content are presented below. Specific adaptations for each of the 3a, 3b, 3c, and 3d categories are listed in Table 2.

**Table 2:** List of adaptations

Judging	Adaptations
Behavioral and environmental fit	Shifting the focus from weight loss to healthy eating and physical activity
	Emphasize the importance of healthy lifestyle on overall HRQoL
	Acknowledge the difficulty of behavior change given the symptoms and side effects burden
	Keep their health care team updated of any weight changes > 5%
	Diet quality: Stronger focus on a plant-based diet (based on vegetables, fruits, whole grains, beans,nuts and limiting the amount of red and processed meat, other processed foods, salt, added sugars, refined grains, and saturated fats)
	Diet quantity: Portion recommendation based on a 2.000 kcal diet. “Cut down on quantity” section rephrased to “Keep an eye on portion sizes” with respective changes in food portion recommendations
	Physical activity: Emphasize the link between physical activity and cancer and its benefits particularly for cancer survivors
	Addition of muscle strengthening exercises
	Addition of section on sleep hygiene
	Update of the suggested cookbooks
	Reference to potentially useful mobile applications
Determinants and change methods fit	Addition of practical applications: • Understanding the risk benefits of supplements • Approval of the booklet content from cancer nurses • Recognize ways of maintaining a balanced diet in the presence of bowel symptoms • Recognize ways of avoiding risk for food born-illnesses • Be prompted to carry wallet-size cards with healthy and unhealthy options in various types of restaurants • Be prompted to identify the triggers for unhealthy eating and generate a strategy to overcome them (e.g. do not skip meals before going to a party to avoid overeating) • Know about the link between physical activity and sleep • Provide pictures of cancer survivors demonstrating sample strength, balance, and flexibility exercises and instructions of how to perform those • Practice some of the resistance exercises during the session following the booklet’s instructions • Be prompted to identify the triggers for sedentary behaviors and generate a strategy to overcome them (e.g. have an alternative PA plan in case of foul weather) • Understand what fatigue is • Select strategies from a list of suggestions to help them overcome fatigue and facilitate physical activity
	Removal of practical applications: • Recognition of the various factors that influence weight and the health consequences of obesity • Differentiate refined and unrefined carbohydrates and low/high GI • Calculate % calories from fat in food and compare it with guidelines • Recognize the value of healthy range of weight rather than an ideal weight and the benefits of gradual weight loss • Set a realistic weight goal (either loss or maintenance) as an outcome of changed eating and physical activity patterns • Receive advice to weight themselves on a regular basis that they will decide upon • Record weight in the diaries • Demonstrate BMI calculation • Apply the BMI to their own weight and height and] compare it with standard cut-offs • Recognize the connection between weight control and PA • Recognize approximate energy expenditure (kcal) for various lifestyle activities
Design, delivery, and cultural fit	Addition of motivational quotes from other cancer survivors Addition of cancer-specific resources (e.g. Macmillan Cancer Support) Addition of recommendations for managing fatigue and bowel issues Addition of a section on healthy lifestyle specifically for endometrial cancer survivors
Implementation fit	Addition of short briefing about the adapted program in the facilitators’ training

#### 3a. Behavioral fit

The original “Shape-Up” manual was developed in line with the recommendations in “The Eatwell Plate” and UK physical activity guidelines. The only major change in the adapted version was the shift of focus from weight loss to healthy eating and physical activity, given the lack of strong evidence for the benefits of intentional weight loss in cancer survival outcomes [31]. This also increased the reach of the intervention.

#### 3b. Determinants and change methods

The targeted behaviors, behavioral determinants, and performance objectives remained the same except the performance objective “Cut down on quantity” which was rephrased to “Keep an eye on portion sizes” with respective changes in food portion recommendations. Based on the content-specific changes, change objectives and practical applications targeting weight management were removed while information on late-treatment effects management, and resistance exercises were added (Table 2). These accommodated the change in the program goal to improving participants’ HRQoL.

#### 3c. Delivery, design, and cultural fit

Most participants found the manual useful, yet detailed and bulky. They were particularly keen on tailoring the information to endometrial cancer survivors. The delivery of the intervention remained unchanged, given the high preference of endometrial cancer survivors for face-to-face contact with the intervention facilitator. Retaining the duration, intensity, and frequency of the program to eight weekly meetings aimed to balance the lack of agreement among survivors for the optimal intervention delivery time.

#### 3d. Implementation fit

The program is currently used within the National Health Service. Its most recent service evaluation demonstrated positive results in terms of adoption of health behaviors. For example, 89% of participants reported higher physical activity, and 90% described being better able to manage ‘triggers’ that may lead to unhealthy behaviors [26].

The implementation fit was considered adequate given the conservation of the initial delivery method. Nonetheless, disease-free endometrial cancer survivors are a very specific segment of the population widely dispersed across the country. Thus, program implementation might be more challenging in local centers, such as community centers, whereas treatment hospitals, and Macmillan Cancer Support Centers might serve this purpose well.

Furthermore, clinicians and researchers participating in the NCRI gynecology workshop were supportive of the program, indicating its future potential acceptability. The dietitian involved, who might represent potential program implementers, was familiar with the original program. Additionally, the intervention is in accordance with the self-help and personalized support envisioned in the National Cancer Survivorship Initiative [32].

#### 3e. Essential elements

Based on the intensity of the BCTs used, those considered essential included self-monitoring of behavior, behavioral goal setting, self-incentives, social support, and problem solving. These were also the core BCTs for facilitating health behavior change identified in the literature [20, 27–29]. The differences in the BCTs between the original and adapted versions are available in the Additional file 1: Table S1. These differences primarily reflect the weight loss component and were not considered essential elements of the program based on the results of the service evaluation that highlighted the focus on feedback regarding behavior changes over weight monitoring [26]. This indicated that the step-by-step approach was comprehensively addressing behavior modification. The three added BCTs were 1) demonstration of behavior for the resistance exercises, 2) conservation of mental resources (which was a refinement of an existing program component), and 3) credible sources for making the program relevant to the targeted population.

It could be argued that the combination of BCTs make the program effective rather than specific individual ones. Furthermore, all BCTs identified in the literature and coded against the BCTTv1 were included in both the original and adapted versions of the program. Therefore, the theoretical methods of the program remained largely unchanged. Examples of the adapted matrices for change objectives for diet and physical activity are shown in Tables 3 and 4, respectively. The full matrices are available in the Additional file 1: Tables S3 and S4.

**Table 3:** Example matrix for change objectives aiming at establishing a healthy diet (behavior)

Performance objectives	Change objectives	BCTs	Theory	Practical application	S/SB/B
PO 1. Have a regular eating pattern	OE 1.1. Identify their current eating pattern	Feedback on behavior	CT	From a list of 7 different eating patterns, the person picks the one that applies to them and reads feedback based on their choices	B
	KN 1.1. Recognize the benefits of regular eating and breakfast	Information about health consequences	SCT	Verbal and written explanation that regular eating can regulate hunger and that breakfast can improve cardio metabolic risk	SB
	SE 1.1. Express confidence in their ability to start following a regular eating pattern and eating breakfast regularly	Habit formation	SCT	Be prompted to start eating at the same time each day	SB
	SE 1.2. Express confidence in eating regularly	Problem solving and social support (unspecified)	SCT	Identify barriers to eating regularly during the previous week	S
				Generate strategies (as a group) to overcome barriers and increase facilitators to regular eating	S
	GO 1.1. Be prompted to increase the difficulty of their goals slowly until behavior is performed	Graded tasks	SCT	Be prompted to make behavioral changes in the following order • Changes towards PO 1 • Changes towards PO 2 • Changes towards PO 3	SB
	GO 1.2. Set a regular eating goal	Behavioral goal setting	SCT	Set a SMART regular eating goal based on GO 4.1.	S

Determinant-specific change objectives target each performance objective (PO). The determinants were outcome expectations (OE), knowledge (KN), self-efficacy (SE), and goals (GO). The BCTs and practical applications are present only in the sessions (S); both in the sessions and the booklet (SB); or only in the booklet (B). BCTs: Behavior Change Techniques, CT: Control Theory, SCT: Social Cognitive Theory

**Table 4:** Example matrix for change objectives aiming at increasing physical activity (behavior)

Performance objectives	Change objectives	BCTs	Theory	Practical application	S/SB/B
PO 1. Reduce sedentary behaviors	OE 1.1. Assess their current PA and evaluate required PA changes	Feedback on behavior	CT	Complete a quiz about physical activity levels and receive written feedback based on their score.	SB
	OE 1.2. Understand the difference between physical activity and exercise	Framing/reframing	CT	Understand that exercise is only one of a range of physical activities that can promote health	S
	KN 1.1. Recognize the benefits of PA	Information about health and emotional consequences	SCT	Know about the physical and emotional benefits of physical activity	SB
				Know about the link between physical activity and sleep	
	SE 1.1. Express confidence in being more physically active	Problem solving	SCT	Identify potential barriers to physical activity and generate strategies to overcome them	SB
	SE 1.2. Express confidence in reducing sedentary behaviors	Instructions on how to perform the behavior	SCT	Recognize ways of reducing sedentary behaviors	B
				Recognize ways of improving sleep quality	B
	GO 1.1. Set a goal to reduce sedentary time	Behavioral goal setting	SCT	Set a SMART physical activity goal based on GO 4.1.	S

Determinant-specific change objectives target each performance objective (PO). The determinants were outcome expectations (OE), knowledge (KN), self-efficacy (SE), and goals (GO). The BCTs and practical applications are present only in the sessions (S); both in the sessions and the booklet (SB); or only in the booklet (B). BCTs: Behavior Change Techniques, CT: Control Theory, SCT: Social Cognitive Theory

### Step 4 Adaptations

The production of the materials was based on the planned changes. The iterative review process ensured an optimal match between program goals and materials. A pilot evaluation of the program has recently been carried out (DEUS; Diet and Exercise in Uterine Cancer Survivors) pilot trial (ClinicalTrials.gov identifier: NCT02433080 [33]. Participants from this pilot were asked to provide rich feedback through open-ended questions about the delivery and the materials of the program upon completion of the intervention. This will be reported in the outcomes paper for this pilot. The final copy of the “Shape-Up following cancer treatment” manual was used for the pilot evaluation (Step 6).

### Step 5 Plan for implementation

#### 5a. Facilitators, facilitation behaviors, and outcomes

For the original program, potential facilitators (allied health care professionals or lay volunteers interested in facilitating group) were trained to follow a scripted manual for delivery of the intervention. While one facilitator was considered sufficient, the presence of a second was also thought appropriate in case of unexpected circumstances. From the facilitators’ perspective, the original implementation protocol was deemed sufficient for adoption in the new setting. This also followed the suggestions from the qualitative work [8]. Therefore, potential facilitators were not involved at this stage.

As geographical proximity was a significant barrier for participation, the pilot intervention took place in one of the recruitment hospitals in central London aiming to provide a location easily accessed by transport. Based on the areas covered by the two recruitment hospitals, it was anticipated that most participants would need to commute less than 45 min each way to attend the face-to-face program.

The service evaluation of the original “Shape-Up” is primarily based on attendance rate and weight measurements. However, weight tracking was deemed inappropriate for the adapted version. Depending on resources, other criteria such as assessments of health status and HRQoL might determine program sustainability at program completion and at least 6-month follow-up. Quality control visits at random sessions was used as a measure of protocol fidelity. The implementation outcomes were expected to be 90% protocol fidelity and at least 60% adherence, as defined by attendance of sessions. These closely matched the observed fidelity and adherence rates of 85% and 61%, respectively.

#### 5b. Facilitation scope, sequence, and instructions

The above similarities between the two program versions led to identical matrices of change objectives for effectively facilitating a group (Additional file 1: Table S5). However, the facilitator’s manual was modified to reflect the changes in the new program and the unique challenges that this population experience based on Table 2. The 158-page manual provides detailed directions on setting up the program, addresses common challenges in group settings, and contains explicit instructions for the delivery of the program. The scope of the program is delivery of eight 90-min sessions once per week for 8 weeks in groups of around eight participants. The order is determined by the sequence of the topics in the program (Additional file 1: Table S4).

#### 5c. Activities for training facilitators

Weight Concern had previously developed a one-day training session for potential facilitators of the original program. This explained the theory of the program, its core elements, quality of delivery, management of challenging situations, and directions for setting up a group. It targeted outcome expectations, knowledge, and self-efficacy. A short briefing explaining the differences between the two programs was added to the original training to enable the training for both programs to be easily merged.

#### 5d. Logistics

The total cost per participant in 2015 was estimated to be £79.33 and £39.33 if a dietician or a trained volunteer, respectively, delivers the intervention (Additional file 1).

### Step 6 Plan for evaluation

The intervention has recently been evaluated within the DEUS (Diet and Exercise in Uterine Cancer Survivors) pilot trial in disease-free survivors within 3 years of diagnosis (ClinicalTrials.gov identifier: NCT02433080) [33, 34].

## Discussion

The current study presents the adaptation process of an evidence-based program focusing on healthy eating and physical activity for endometrial cancer survivors. The use of the IM systematic framework for its adaptation, implementation, and evaluation increases the potential for intervention effectiveness. The systematic process ensured all potential intervention aspects were considered. It described comprehensively the link between theory, BCTs and practice, helping interventionists understand the unique value of each essential intervention element and their interrelationship.

As, it is currently unclear which interventions are most effective, use of theory for the development and implementation of behavior change interventions has been proposed as a means of increasing their effectiveness, and provide insights into the mediators of behavior change that can, subsequently, translate to successful interventions [35]. SCT is one of the most widely used theoretical models for health promotion both in the general population and in cancer survivors as it helps address the mechanisms to change behavior, such as strengthening the self-efficacy belief and facilitating self-management skills [18]. It is also in line with the National Cancer Survivorship Initiative, which recommends self-management as an integral part of survivorship care, including lifestyle changes [36]. Lifestyle interventions based on SCT have yielded promising outcomes in cancer survivors including endometrial cancer [9]. However, the SCT constructs associated with their effectiveness are, at best, inadequately reported [12].

If successful lifestyle interventions are to shape practice, they require suitable characterization methodology and connection to an analysis of the behavior. The IM framework has been used widely for the development of health promotion programs, including nutrition and physical activity [13]. This is the first report of a systematic development of a healthy eating and physical activity program in endometrial cancer survivors. Only one program has been reported in the literature mentioning its development through a systematic framework. The “Kanker Nazorg Wijzer (Cancer Aftercare Guide)” intervention was a web-based holistic intervention addressing psychosocial issues and smoking together with healthy eating and physical activity in cancer survivors. It was also developed using the IM approach and targeted similar behavioral determinants to the current intervention (i.e. self-efficacy, outcome expectations, knowledge) [37]. The intervention resulted in significant changes only in vegetable intake among the dietary behaviors and in moderate physical activity among physical activity behaviors but the study was not powered on these outcomes [38]. Another strength of the current study was that the provided dietary advice is consistent with the newly released dietary recommendations (the “The Eatwell Guide”) which replaced “The Eatwell Plate” a year after the intervention development [39].

Limitations of this study include the retrospective evaluation of the original “Shape-Up”, and, potentially, the use of many BCTs that have not been identified in systematic reviews (Additional file 1: Table S1). However, the lack of evidence might stem from the lack of use of these BCTs or poor reporting rather than evidence of absence of their effectiveness. Furthermore, the combination of all those BCTs seems to make “Shape-Up” effective, rather than each specific BCT given the multiple performance objectives it addresses. IM Adapt was a complex process requiring familiarity with behavioral theory but its systematic approach can ensure better intervention reporting which can lead to better understanding of effective interventions. Moreover, it is generally recognized that multi-level interventions are needed for health behavior changes to be efficacious in the long-term. While the current model comprehensively addresses individual behavioral determinants, it does not consider the environment factors influencing behavior. Therefore, incorporation of the program in a framework of multi-level interventions could comprehensively address health promotion policy efforts. One such framework is the NOURISHING framework developed by the World Cancer Research Fund [40]. The intervention fits well under the section “Nutrition advice and counseling in health care settings”. Additionally, the COM-B model could have been a new alternative systematic framework for analyzing this intervention [41] but its lack of extensive field-testing rendered its application cautionary.

## Conclusions

In conclusion, systematic intervention mapping provided a framework to design a cancer survivor-centered lifestyle intervention. Survivors welcomed the intervention and provided essential feedback for its adaptation. The program has recently been evaluated through a pilot randomized controlled trial. Results of this trial will inform refinements of the intervention and further testing to determine the program’s effectiveness.

## Additional files

Additional file 1: Full matrices of change objectives for healthy eating and physical activity, program structure, and additional methodology. Methodology for searching for evidence-based interventions; Methodology for the qualitative interviews; **Table S1.** BCTs in the original and adapted program versions and effective BCTs from the literature; **Table S2.** Matrix for change objectives aiming at establishing a healthy diet; **Table S3.** Matrix for change objectives aiming to increase physical activity to the extent of at least meeting the PA guidelines; **Table S4.** Structure and content of the “Shape-up following cancer treatment” sessions; **Table S5.** Matrix for change objectives for effectively facilitating a group session (behavior). (DOCX 112 kb)

## Abbreviations

BCT: Behavior Change Technique
COM-B: Capability, opportunity, motivation, behavior
DEUS: Diet and Exercise in Uterine Cancer Survivors
EBI: Evidence-based interventions
HRQoL: Health-related quality of life
IM: Intervention mapping
NCRI: National Cancer Research Institute
SCT: Social Cognitive Theory

## References

[1] Ward KK, Shah NR, Saenz CC, McHale MT, Alvarez EA, Plaxe SC (2012). Cardiovascular disease is the leading cause of death among endometrial cancer patients. Gynecol Oncol.

[2] Leach CR, Weaver KE, Aziz NM, Alfano CM, Bellizzi KM, Kent EE, Forsythe LP, Rowland JH (2015). The complex health profile of long-term cancer survivors: prevalence and predictors of comorbid conditions. J Cancer Surviv.

[3] Koutoukidis DA, Knobf MT, Lanceley A (2015). Obesity, diet, physical activity, and health-related quality of life in endometrial cancer survivors. Nutr Rev.

[4] Secord AA, Hasselblad V, Von Gruenigen VE, Gehrig PA, Modesitt SC, Bae-Jump V, Havrilesky LJ (2016). Body mass index and mortality in endometrial cancer: a systematic review and meta-analysis. Gynecol Oncol.

[5] Clark LH, Ko EM, Kernodle A, Harris A, Moore DT, Gehrig PA, Bae-Jump V (2016). Endometrial Cancer Survivors' perceptions of provider obesity counseling and attempted behavior change: are we seizing the moment?. Int J Gynecol Cancer.

[6] Rock CL, Doyle C, Demark-Wahnefried W, Meyerhardt J, Courneya KS, Schwartz AL, Bandera EV, Hamilton KK, Grant B, McCullough M (2012). Nutrition and physical activity guidelines for cancer survivors. CA Cancer J Clin.

[7] McBride CM, Clipp E, Peterson BL, Lipkus IM, Demark-Wahnefried W (2000). Psychological impact of diagnosis and risk reduction among cancer survivors. Psychooncology.

[8] Koutoukidis DA, Beeken RJ, Lopes S, Knobf MT, Lanceley A. Attitudes, challenges, and needs about diet and physical activity in endometrial cancer survivors: a qualitative study. Eur J Cancer Care (Engl). 2016;26(6). 10.1111/ecc.12531 27324208

[9] von Gruenigen V, Frasure H, Kavanagh MB, Janata J, Waggoner S, Rose P, Lerner E, Courneya KS (2012). Survivors of uterine cancer empowered by exercise and healthy diet (SUCCEED): a randomized controlled trial. Gynecol Oncol.

[10] von Gruenigen VE, Courneya KS, Gibbons HE, Kavanagh MB, Waggoner SE, Lerner E (2008). Feasibility and effectiveness of a lifestyle intervention program in obese endometrial cancer patients: a randomized trial. Gynecol Oncol.

[11] Harrigan M, Cartmel B, Loftfield E, Sanft T, Chagpar AB, Zhou Y, Playdon M, Li F, Irwin ML (2016). Randomized trial comparing telephone versus in-person weight loss counseling on body composition and circulating biomarkers in women treated for breast Cancer: the lifestyle, exercise, and nutrition (LEAN) study. J Clin Oncol.

[12] Stacey FG, James EL, Chapman K, Courneya KS, Lubans DR (2015). A systematic review and meta-analysis of social cognitive theory-based physical activity and/or nutrition behavior change interventions for cancer survivors. J Cancer Surviv.

[13] Bartholomew LK, Parcel GS, Kok G, Gottlieb NH, Fenrnandez ME (2011). Planning health promotion programs: an intervention mapping approach.

[14] Highfield L, Hartman MA, Mullen PD, Rodriguez SA, Fernandez ME, Bartholomew LK. Intervention mapping to adapt evidence-based interventions for use in practice: increasing mammography among African American women. 2015;2015 10.1155/2015/160103 PMC4637430 26587531

[15] Wardle J, Liao LM, Rapoport L, Hillsdon M, Crocker H, Edwards C (2013). Shape-up: a lifestyle programme to manage your weight.

[16] Chadwivk P, Miller S. Shape-up groups: group Facilitator's manual. 3rd ed: Weight Concern; 2014.

[17] Rapoport L, Clark M, Wardle J (2000). Evaluation of a modified cognitive-behavioural programme for weight management. Int J Obes Relat Metab Disord.

[18] Bandura A (2004). Health promotion by social cognitive means. Health Educ Behav.

[19] Carver CS, Scheier MF (1982). Control theory: a useful conceptual framework for personality-social, clinical, and health psychology. Psychol Bull.

[20] Michie S, Abraham C, Whittington C, McAteer J, Gupta S (2009). Effective techniques in healthy eating and physical activity interventions: a meta-regression. Health Psychol.

[21] Prestwich A, Sniehotta FF, Whittington C, Dombrowski SU, Rogers L, Michie S (2014). Does theory influence the effectiveness of health behavior interventions? Meta-analysis. Health Psychol.

[22] Michie S, Richardson M, Johnston M, Abraham C, Francis J, Hardeman W, Eccles MP, Cane J, Wood CE (2013). The behavior change technique taxonomy (v1) of 93 hierarchically clustered techniques: building an international consensus for the reporting of behavior change interventions. Ann Behav Med.

[23] Hoffmann TC, Glasziou PP, Boutron I, Milne R, Perera R, Moher D, Altman DG, Barbour V, Macdonald H, Johnston M (2014). Better reporting of interventions: template for intervention description and replication (TIDieR) checklist and guide. BMJ.

[24] Krueger R. Pilot testing new materials: developing questions for focus groups. In: The focus group kit. London: SAGE Publications; 1997. p. 97.

[25] Braun V, Clarke V (2006). Using thematic analysis in psychology. Qualit Res Psychol.

[26] ACE (2013). Shape-up annual report for 2011/2012. Colchester: Anglian community Enterprise, weight concern, and national health service.

[27] French D, Olander E, Williams S, Fletcher H, Atkinson L, Turner A: Building an evidence base for skills development training for cancer clinicians to support lifestyle behaviour change and self-management with cancer survivors. In: National Cancer Survivorship. Coventry: Applied Research Centre in Health & Lifestyle Interventions, Faculty of Health & Life Sciences, Coventry University; 2011.

[28] Treating adult obesity through lifestyle change interventions: A briefing paper for commissioners [http://www.noo.org.uk/uploads/doc/vid_5189_Adult_weight_management_Final_220210.pdf]. Accessed 1 Dec 2017.

[29] NICE (2014). Managing overweight and obesity in adults – lifestyle weight management services.

[30] Cocks K, King MT, Velikova G, de Castro G, Martyn St-James M, Fayers PM, Brown JM (2012). Evidence-based guidelines for interpreting change scores for the European organisation for the research and treatment of Cancer quality of life questionnaire Core 30. Eur J Cancer.

[31] Jackson SE, Heinrich M, Beeken RJ, Wardle J (2017). Weight loss and mortality in overweight and obese Cancer survivors: a systematic review. PLoS One.

[32] DoH, Department of Health MCSNI (2010). National Cancer Survivorship Initiative Vision.

[33] Koutoukidis DA, Beeken RJ, Manchanda R, Burnell M, Knobf MT, Lanceley A (2016). Diet and exercise in uterine cancer survivors (DEUS pilot) - piloting a healthy eating and physical activity program: study protocol for a randomized controlled trial. Trials.

[34] Koutoukidis DA, Beeken RJ, Manchanda R, Michalopoulou M, Burnell M, Knobf MT, Lanceley A (2017). Recruitment, adherence, and retention of endometrial cancer survivors in a behavioural lifestyle programme: the diet and exercise in uterine Cancer survivors (DEUS) parallel randomised pilot trial. BMJ Open.

[35] Glanz K, Bishop DB (2010). The role of behavioral science theory in development and implementation of public health interventions. Annu Rev Public Health.

[36] DoH. Living with & beyond Cancer: taking action to improve outcomes (an update to the 2010 the National Cancer Survivorship Initiative Vision). London: Department of Health, Macmillan Cancer Support & NHS Improvement; 2013.

[37] Willems RA, Bolman CA, Mesters I, Kanera IM, Beaulen AA, Lechner L (2015). The Kanker Nazorg Wijzer (Cancer aftercare guide) protocol: the systematic development of a web-based computer tailored intervention providing psychosocial and lifestyle support for cancer survivors. BMC Cancer.

[38] Kanera IM, Bolman CA, Willems RA, Mesters I, Lechner L. Lifestyle-related effects of the web-based Kanker Nazorg Wijzer (Cancer aftercare guide) intervention for cancer survivors: a randomized controlled trial. J Cancer Surviv. 2016; 10.1007/s11764-016-0535-6 PMC5018034 26984534

[39] PHE (2016). The Eatwell guide: how does it differ to the eatwell plate and why?.

[40] NOURISHING framework [http://www.wcrf.org/int/policy/nourishing-framework]. Accessed 1 Dec 2017.

[41] Michie S, van Stralen MM, West R (2011). The behaviour change wheel: a new method for characterising and designing behaviour change interventions. Implement Sci.

