# Supplementary material for: Use of intervention mapping to adapt a health behavior change intervention for endometrial cancer survivors: the shape-up following cancer treatment program
Source: BMC Public Health. 2018 Mar 27;18:415. doi: 10.1186/s12889-018-5329-5 (PMC5869761; doi:10.1186/s12889-018-5329-5)
Supplement: Supplementary file 1 — Full matrices of change objectives for healthy eating and physical activity, program structure, and additional methodology. Methodology for searching for evidence-based interventions; Methodology for the qualitative interviews; Table S1. BCTs in the original and adapted program versions and effective BCTs from the literature; Table S2. Matrix for change objectives aiming at establishing a healthy diet; Table S3. Matrix for change objectives aiming to increase physical activity to the extent of at least meeting the PA guidelines; Table S4. Structure and content of the “Shape-up following cancer treatment” sessions; Table S5. Matrix for change objectives for effectively facilitating a group session (behavior). (DOCX 112 kb) [file 12889_2018_5329_MOESM1_ESM.docx]

**Additional file 1**

**Use of intervention mapping to adapt a health behavior change intervention for endometrial cancer survivors: the Shape-Up following cancer treatment program**

Dimitrios A. Koutoukidis, Sonia Lopes, Helen Croker, Lou Atkins, M. Tish Knobf, Anne Lanceley and Rebecca J. Beeken

**Step 2: Methodology for searching for evidence-based interventions**

PubMed and the Internet were searched for available self-help materials on diet, nutrition, and physical activity in cancer survivors using relevant search terms. Two databases of effective interventions were also searched using criteria for healthy eating, physical activity, or survivorship programs in adults that were tested in a randomized controlled trial with at least two follow-up assessments. These included the National Cancer Institute Research-tested Intervention Programs database and the Centre for Training and Research Translation database. The grey literature search included the lifestyle guidelines for cancer survivors by key organisations; the World Cancer Research Fund, the American Cancer Society, and the American College of Sports Medicine [1-3].

**Step 3: Methodology for the qualitative interviews**

In order to judge the delivery, design, and cultural fit, DAK interviewed 15 endometrial cancer survivors who participated in a previous qualitative study conducted by the research team [4]. Other than the previous study, the interviewer did not have any previous relationship with the study participants. The survivors were asked to review the original Shape-Up booklet for a week prior to the interview. Each 15-minute telephone interview was audio-recorded and semi-structured. The interview guide is shown on Table S6. Data were transcribed verbatim and listened to for accuracy verification. Two coders (DAK, SL) analyzed the data using the six-phase thematic analysis [5] in NVivo version 10 (QSR International Pty Ltd, 2014). Themes were identified at an explicit level using a realist approach. Following familiarization with the data. DAK generated a coding framework by coding the first four interviews using an inductive approach. SL second-coded the first-four interviews to ensure inter-rater reliability. The rest of the transcripts were then coded. Individual suggestions for improvements in the manual were divided in two themes (content-specific and format-specific). Because each participant had different suggestions, all suggestions were reported to maximize the acceptability of the manual to cancer survivors.

**Step 5d: Logistics**

The estimated printing cost for one “Shape-Up following cancer treatment” manual was £14.95 (exclusive of VAT) based on a bulk order of 100 manuals in 2015. The remaining logistics were similar to the original program with the facilitators’ training to cost £195 per trainee in 2015, inclusive of the facilitator’s manual. Taking into account the facilitator’s training, booklet production, and dietitian’s salary (£20 per hour, 2 hours per session) and assuming 8 participants per group session and free hiring of a community space, the cost of the intervention was estimated to be £9.92 per participant per session or £79.33 per participant for the whole program. Delivery of the intervention by trained volunteers halves the program cost (£39.33 per participant).

**List of tables**

[Table S1 BCTs in the original and adapted program versions and effective BCTs from the literature 3](#_Toc503939460)

[Table S2 Matrix for change objectives aiming at establishing a healthy diet (behaviour) 6](#_Toc503939461)

[Table S3 Matrix for change objectives aiming to increase physical activity to the extent of at least meeting the PA guidelines (behaviour) 18](#_Toc503939462)

[Table S4 Structure and content of the “Shape-up following cancer treatment” sessions 26](#_Toc503939463)

[Table S5 Matrix for change objectives for effectively facilitating a group session (behavior) 30](#_Toc503939464)

[Table S6 Interview guide about the “Shape-Up” program (Adapted from [8]) 33](#_Toc503939465)

Table S1 BCTs in the original and adapted program versions and effective BCTs from the literature

|  |  | Behaviors in original “Shape-Up” | | Behaviors in “Shape-Up following cancer treatment” | | BCTs identified from the literature to facilitate health behavior changes | | |
| --- | --- | --- | --- | --- | --- | --- | --- | --- |
| BCT Category | BCT | Establish a healthy diet | Increase physical activity to the extent of at least meeting the PA guidelines | Establish a healthy diet | Increase physical activity to the extent of at least meeting the PA guidelines | Systematic reviews [6, 7] | NOO Guidelines [8] | NICE guidelines [9] |
| Goals and planning | Behavioral goal setting |  |  |  |  |  |  |  |
|  | Problem solving |  |  |  |  |  |  |  |
|  | Action planning |  |  |  |  |  |  |  |
|  | Review behavioral goals |  |  |  |  |  |  |  |
|  | Discrepancy between current behavior and goal |  |  |  |  |  |  |  |
|  | Goal setting (outcome)^a^ |  |  |  |  |  |  |  |
| Feedback and monitoring | Self-monitoring of behavior |  |  |  |  |  |  |  |
|  | Self-monitoring of outcome of behavior^a^ |  |  |  |  |  |  |  |
|  | Feedback on behavior |  |  |  |  |  |  |  |
|  | Feedback on outcome of behavior^a^ |  |  |  |  |  |  |  |
| Social support | Social support (unspecified) |  |  |  |  |  |  |  |
| Shaping knowledge | Instructions on how to perform behavior |  |  |  |  |  |  |  |
| Natural consequences | Information about health consequences |  |  |  |  |  |  |  |
|  | Information about emotional consequences |  |  |  |  |  |  |  |
| Comparison of behavior | Demonstration of behavior^b^ |  |  |  |  |  |  |  |
|  | Facilitate social comparison |  |  |  |  |  |  |  |
|  | Information about others’ approval |  |  |  |  |  |  |  |
| Associations | Satiation^a^ |  |  |  |  |  |  |  |
| Repetition and substitution | Behavioral practice |  |  |  |  |  |  |  |
|  | Behavior substitution |  |  |  |  |  |  |  |
|  | Habit formation |  |  |  |  |  |  |  |
|  | Graded tasks |  |  |  |  |  |  |  |
| Comparison of outcomes | Credible source^b^ |  |  |  |  |  |  |  |
|  | Pros and cons |  |  |  |  |  |  |  |
| Reward and threat | Social reward |  |  |  |  |  |  |  |
|  | Self-incentive |  |  |  |  |  |  |  |
|  | Non-specific incentive |  |  |  |  |  |  |  |
| Regulation | Reduce negative emotions |  |  |  |  |  |  |  |
|  | Conserving mental resources^b^ |  |  |  |  |  |  |  |
| Antecedents | Reducing exposure to cues for the behavior |  |  |  |  |  |  |  |
|  | Distraction |  |  |  |  |  |  |  |
|  | Information about antecedents |  |  |  |  |  |  |  |
| Identity | Framing / reframing |  |  |  |  |  |  |  |
| Self-belief | Verbal persuasion about capabilities |  |  |  |  |  |  |  |
|  | Self-talk |  |  |  |  |  |  |  |
| Non-categorized | Time management |  |  |  |  |  |  |  |
|  | Use of follow-up prompts |  |  |  |  |  |  |  |
|  | Assertiveness |  |  |  |  |  |  |  |
| ^a^: BCTs removed from the original Shape-Up; ^b^: BCTs added in the adapted version. All BCTs are based on the BCTTv1 taxonomy apart from the non-categorized that are based on the CALO-RE taxonomy. NOO: National Obesity Observatory. | | | | | | | | |

Table S2 Matrix for change objectives aiming at establishing a healthy diet (behaviour)

| **Performance objectives** | **Change objectives** | **BCTs** | **Theory** | **Practical application** | **S/SB/B** |
| --- | --- | --- | --- | --- | --- |
| PO 1. Have a regular eating pattern | OE 1.1. Identify their current eating pattern | Feedback on behavior | CT | From a list of 7 different eating patterns, the person picks the one that applies to them and reads feedback based on their choices | B |
|  | KN 1.1. Recognize the benefits of regular eating and breakfast | Information about health consequences | SCT | Verbal and written explanation that regular eating can regulate hunger and that breakfast can improve cardio metabolic risk | SB |
|  | SE 1.1. Express confidence in their ability to start following a regular eating pattern and eating breakfast regularly | Habit formation | SCT | Be prompted to start eating at the same time each day | SB |
|  | SE 1.2. Express confidence in eating regularly | Problem solving and social support (unspecified) | SCT | Identify barriers to eating regularly during the previous week | S |
|  |  |  |  | Generate strategies (as a group) to overcome barriers and increase facilitators to regular eating | S |
|  | GO 1.1. Be prompted to increase the difficulty of their goals slowly until behavior is performed | Graded tasks | SCT | Be prompted to make behavioral changes in the following order   - Changes towards PO 1 - Changes towards PO 2 - Changes towards PO 3 | SB |
|  | GO 1.2. Set a regular eating goal | Behavioral goal setting | SCT | Set a SMART regular eating goal based on GO 4.1. | S |
| PO 2. Adhere to the Eatwell plate principles | OE 2.1. Recognize the value of the behavioral recommendations | Framing / reframing | CT | Identify the difference between a fad diet and learning how to carry on with the healthy lifestyle for a lifetime | B |
|  | OE 2.2. Review their shopping pattern | Feedback on behavior | CT | From a list of 7 different shopping patterns, the person picks the one that applies to them and reads feedback based on their choices | B |
|  | OE 2.3. Be aware that others also face difficulties following a healthy diet after cancer treatment and increase motivation to be more active | Information about others’ approval | SCT | Read motivational quotes from other cancer survivors about healthy eating | B |
|  | OE 2.3. Increase expectation that health care professionals will approve their lifestyle changes | Credible source | SCT | Read information from nurses in favor of healthy eating | B |
|  | KN 2.1. Understand components of each food group and their benefits and risks | Information about health consequences | SCT | Explanation of the health benefits (vegetables, fruits, whole grains, pulses, healthy oils) and risks of the various food groups (foods/drinks high in fat/sugar, saturated and trans fats, refined grains, red meat, excessive alcohol intake) | SB |
|  |  |  |  | Recognize the principles of Eatwell plate, the five food groups and their ideal proportions | SB |
|  |  |  |  | Recognize the different names of sugar and fat on food labels | SB |
|  |  |  |  | Understand the risks and benefits of supplements | SB |
| PO 2. Adhere to the Eatwell plate principles (cont’d) | SE 2.1. Express confidence in choosing healthy foods and drinks | Instructions on how to perform the behavior | SCT | Recognize ways of eating enough fruits, vegetables and whole grains; making healthy choices from the protein & dairy groups; eating less fatty and sugary foods; and cutting down alcohol | SB |
|  |  |  |  | Recognize ways of cutting down on sugary drinks | B |
|  |  |  |  | Recognize ways of having healthier lunches | B |
|  |  |  |  | Recognize which snack to choose | B |
|  |  |  |  | Identify foods to store in the cupboard / fridge / freezer | B |
|  |  |  |  | List ways that eating out can fit into the new eating plan | B |
|  |  |  |  | Recognize healthy and unhealthy options in various types of restaurants | B |
|  |  |  |  | Recognize healthy options in ready meals | B |
|  |  |  |  | Recognize ways of having an affordable healthy diet | B |
|  |  |  |  | Consult when necessary suggested cookbooks | B |
|  |  | Behavioral practice / rehearsal | SCT | Practice matching various foods under each food group | S |
|  | SE 2.2. Express confidence in maintaining a balanced diet in the presence of bowel symptoms | Instructions on how to perform the behavior | SCT | Recognize ways of maintaining a balanced diet in the presence of bowel symptoms | B |
| PO 2. Adhere to the Eatwell plate principles (cont’d) | SE 2.3. Express confidence in food safety | Instructions on how to perform the behavior | SCT | Recognize ways of avoiding risk for food born-illnesses | B |
|  | SE 2.4. Express confidence in adhering to the Eatwell plate when eating out | Conserving mental resources | SCT | Be prompted to carry wallet-size cards with healthy and unhealthy options in various types of restaurants | B |
|  | SE 2.5. Express confidence in understanding food labels | Behavioral practice/rehearsal | SCT | Examine the ingredients list | SB |
|  |  |  |  | Compare nutritional values of various sweet and savory snacks | B |
|  |  |  |  | Compare different product labels to the guidelines and to the guide to good shopping | SB |
|  |  |  |  | Examine the sugar, fat, and calorie content per 100g, compare it to guidelines, and decide if the foods are healthy options | SB |
|  |  |  |  | Interpret nutritional claims correctly | B |
|  | GO 2.1. Set a healthy eating goal | Behavioral goal setting | SCT | Set one or more SMART goal on eating a balanced diet based on GO 4.1. | S |
| PO 3. Keep an eye on portion sizes | OE 3.1. Recognize their aims regarding food quantity | Information about health consequences | SCT | Written explanation that keeping an eye on portion sizes can help them avoid undesirable weight gain | B |
|  | KN 3.1. Understand what are the recommended amounts from each food group to avoid weight gain | Instructions on how to perform the behavior | SCT | Describe what constitutes a serving from each group | S |
|  |  |  |  | Describe the amounts of servings aiming for from each group | S |
|  |  |  |  | Observe portion sizes of various foods from all food groups | SB |
| PO 3. Keep an eye on portion sizes (cont’d) | SE 3.1. Express confidence in keeping an eye on portion sizes | Behavioral practice/rehearsal | SCT | Using the food portions, create a day’s food intake aligned with the recommendations | S |
|  | GO 3.1. See GO 4.1 |  |  |  |  |
| PO 4. Create and maintain a discrepancy-reducing feedback loop | OE 4.1. Express confidence in their ability to change their behavior | Pros and cons | SCT | Explain of the components of motivation to change | SB |
|  |  |  |  | Review examples of advantages and disadvantages for (not) changing their lifestyle | SB |
|  |  |  |  | Analyze their advantages and disadvantages of (not) changing their lifestyle | SB |
|  | OE. 4.2. Recognize the importance of making one behavior change at a time | Framing / reframing | CT | Recognize that making one behavior change at a time will be more advantageous in the long term compared to making too many changes at once | SB |
|  | OE 4.3. Recognize the value of self-monitoring | Self-monitoring of behavior | CT | Written explanation that self-monitoring of behavior can improve behavior practice and maintenance | SB |
|  |  |  |  | Provision of food diaries each week | SB |
|  |  |  |  | Record the time, amount and type of food and drink each day using (if preferred) the Shape-Up diary | SB |
|  |  |  |  | Verbal re-affirmation that self-monitoring of behavior through diaries can improve behavior practice and maintenance at the end of the program | S |
| PO 4. Create and maintain a discrepancy-reducing feedback loop (cont’d) | OE 4.5. Express confidence in (not) seeking social support | Problem solving and social support (unspecified) | SCT | Identify helpful and unhelpful social support towards healthy eating changes (e.g. family, friends, health care professionals) | B |
|  |  |  |  | Problem solve potential social impediments | B |
|  | OE 4.6. Plan their self-reward | Self-incentive & non-specific incentive | SCT | Understand the value of rewards for achieving their SMART goals | SB |
|  |  |  |  | Brainstorm ideas for non-food incentives | S |
|  |  |  |  | Plan to reward self when they review their goals if they achieve their SMART goals^b^ | SB |
|  | OE 4.7 Reward success | Social reward | SCT | Facilitator verbally praises participants if and only if participants have progressed with their goals | S |
|  | OE 4.8. Receive support from each other while making behavioral changes | Social support (unspecified) | SCT | Distribute to each other their contact details so that they support each other on the behavioral changes during the program | S |
|  |  |  |  | Be aware that others also face difficulties following a healthy lifestyle after cancer treatment | S |
|  |  |  |  | Get support and ideas from group members on following a healthy lifestyle | S |
|  | OE 4.9. Identify potential sources of external support at the end of the program | Social support (unspecified) | SCT | Discuss if continuous support from other healthy lifestyle services can be helpful after the end of the program | S |
|  |  |  |  | Advice on how they can find information about support in their local area | S |
| PO 4. Create and maintain a discrepancy-reducing feedback loop (cont’d) | KN 4.1. Become aware of their current dietary pattern | Self-monitoring of behavior | CT | Record the time, amount and type of food and drink each day using (if preferred) the Shape-Up diary | B |
|  | SE 4.1. Express confidence in their ability to increase their willpower | Verbal (and written) persuasion about capabilities | SCT | Understand that willpower is a practicable skill (written) | S |
|  |  |  |  | Understand that they can learn to swap unhealthy habits to healthy ones | SB |
|  | SE 4.2. Express confidence in setting SMART healthy eating goals | Behavioral practice | SCT | Define the principles of SMART goal setting | SB |
|  |  |  |  | Practise translating vague goals to SMART ones | SB |
|  | SE 4.3. Express confidence in rewarding themselves for achieving their SMART goals | Self-incentive & non-specific incentive | SCT | List their own potential rewards | B |
|  | SE 4.5. Decide if discrepancies exist when comparing intake to personal goals and guidelines | Discrepancy between current behavior and goal | CT | Compare their score (SE 5.2) to personal goals or guidelines in order to draw attention to the discrepancy | B |
|  | SE 4.6 Maintain self-monitoring through diaries | Problem solving | SCT | Identify as a group barriers to keeping a food and activity diary | S |
|  |  |  |  | Discuss ways in which they can overcome the barriers | S |
|  | SE 4.7. Express confidence in achieving SMART goals | Review of behavioral goals, problem solving, and social support (unspecified) | SCT | Re-examine their behaviors. Facilitator and group members provide evaluative feedback if they have (not) met the SMART criteria and if they have rewarded themselves for achieving the goal. Participants should problem-solve themselves with the help of the rest of the group ways of making their goals fit with the SMART criteria | S |
| PO 4. Create and maintain a discrepancy-reducing feedback loop (cont’d) | GO 4.1. Compose an action plan towards establishing a healthy diet | Action planning and goal setting (behavior) | SCT | Plan in detail the performance of their first SMART goal | SB |
|  |  |  |  | Plan things that might get in the way of achieving the goal | SB |
|  |  |  |  | Plan how and when they will review the goal | SB |
|  |  |  |  | Plan who might help them | SB |
|  | GO 4.2. Set a lifestyle target | Goal setting (outcome) | SCT | Set a lifestyle goal (e.g. healthier life) as an outcome of changing eating and activity behaviors | S |
|  | GO 4.3. Compare intake to personal goals and guidelines | Self-monitoring of behavior and goal setting (behavior) | CT | Categories consumed foods and drinks in servings from each food group | B |
|  |  |  |  | Score their daily consumption of each food group | B |
|  |  |  |  | Review their SMART goal | B |
|  |  |  |  | Evaluate outcome of their SMART goal | B |
|  | GO 4.4. Expressing confidence in maintaining monitoring, goal setting, incentivization after the end of the program | Action planning, goal setting (behavior), problem solving, and self-incentive | SCT | Plan in detail their dietary and physical activity SMART goals, identify barriers and facilitators and strategies to facilitate/overcome them, specify review date, and reward | SB |
|  |  | Review behavior goals and information about others’ approval | SCT | As a group, review each participant’s goals and discuss other’s views on the plan and potential modifications if goals unrealistic or participants overwhelmed | SB |
| PO 5. Deal effectively with triggers of unhealthy eating and lapses | OE 5.1. Reframe unhelpful thoughts about lapses to helpful ones | Framing/reframing | CT | Identify five helpful and four unhelpful example thoughts about lapses from their healthy eating and physical activity plan | S |
|  |  |  |  | For the unhelpful thoughts, generate alternatives that are more positive and/or would help them recover from the lapse | S |
|  | SE 5.1. Express confidence in dealing with external triggers | Avoidance/reducing exposure to cues for the behavior | CT | Be prompted to identify and try to avoid cues promoting unhealthy eating (e.g. sight or smell of food) | SB |
|  |  | Distraction | CT | Be prompted to use an alternative focus for attention to avoid triggers for comfort eating | SB |
|  |  | Problem solving | SCT | Be prompted to identify the triggers for unhealthy eating and generate a strategy to overcome them (e.g. do not skip meals before going to a party to avoid overeating) | SB |
|  |  |  |  | Analyze various example external triggers (e.g. buffet party) and select strategies (i.e. avoidance, distraction, problem solving) to overcome them | S |
|  | SE 5.2. Express confidence in dealing with cravings | Problem solving & distraction | SCT | Identify example eating situations either as hunger or craving | SB |
|  |  |  |  | Receive advice to eat regularly to deal with hunger and information that cravings last for only 20 minutes | SB |
|  |  |  |  | Generate strategies to overcome cravings (e.g. distraction) | S |
| PO 5. Deal effectively with triggers of unhealthy eating and lapses (cont’d) |  |  |  | Be prompted to distract self when a craving comes | SB |
|  | SE 5.3. Express confidence in dealing with unhelpful thoughts | Problem solving | SCT | Understand how unhelpful thoughts trigger unhealthy behaviors | S |
|  |  |  |  | Identify unhelpful thoughts that trigger unhealthy eating behaviors | S |
|  |  |  |  | Generate and select strategies to overcome unhelpful thoughts | S |
|  |  | Behavioral practice | SCT | Practice translating unhelpful thoughts that might impede healthy eating goals to helpful ones | SB |
|  |  | Self-talk | SCT | When experiencing an unhelpful thought, be prompted to remind themselves what they are trying to achieve and why | SB |
|  | SE 5.5. Express confidence in dealing with emotions | Problem solving | SCT | Understand how emotions can trigger unhealthy behaviors | S |
|  |  |  |  | Identify emotions that trigger unhealthy eating behaviors | S |
|  |  |  |  | Generate and select strategies to overcome them | S |
|  |  | Distraction | CT | Identify ways for distracting from the mood | SB |
|  |  |  |  | Be prompted to focus attention on this alternative way when in the mood | SB |
|  |  | Behavioral substitution | CT | Be prompted to substitute screen time with other neutral or positive behaviors | SB |
| PO 5. Deal effectively with triggers of unhealthy eating and lapses (cont’d) |  | Reduce negative emotions | SCT | Be prompted to ask themselves whether eating will change the problem/emotion they currently facing | SB |
|  | SE 5.6. Express confidence in dealing with lapses | Verbal persuasion about capability | SCT | Facilitator to mention that lapses are a normal part of the process of change and that they can still make healthier choices as long as they learn how to deal with the lapses | S |
|  |  | Information about antecedents | SCT | Identify the lapse example (eating biscuits) among the factors (social and environmental events, emotions, and cognitions) that lead to the lapse and its consequences | SB |
|  |  |  |  | Put a chain of antecedents (events, emotions, cognitions), lapse (unhealthy eating, social event), and consequences in the right order | S |
|  |  | Problem solving | SCT | Analyze the above factors, lapse, and consequences, and generate strategies to overcome them | S |
|  |  | Verbal persuasion about capability | SCT | Facilitator to reaffirm that participants can successfully overcome future lapses by reflecting on previous ones | S |
|  |  | Reduce negative emotions | SCT | Distinguish a behavioral lapse from a relapse | SB |
|  |  |  |  | Receive advice on the use of a 5-step cognitive strategy to reduce negative emotions in order to deal successfully with a lapse | SB |
|  | GO 5.1. See GO 4.1 |  |  |  |  |
| Determinant-specific change objectives target each performance objective (PO). The determinants were outcome expectations (OE), knowledge (KN), self-efficacy (SE), and goals (GO). The BCTs and practical applications are present only in the sessions (S); both in the sessions and the booklet (SB); or only in the booklet (B). BCTs: Behavior Change Techniques, CT: Control Theory, SCT: Social Cognitive Theory. | | | | | |

Table S3 Matrix for change objectives aiming to increase physical activity to the extent of at least meeting the PA guidelines (behaviour)

| **Performance objectives** | **Change objectives** | **BCTs** | **Theory** | **Practical application** | **S/SB/B** |
| --- | --- | --- | --- | --- | --- |
| PO 1. Reduce sedentary behaviors | OE 1.1. Assess their current PA and evaluate required PA changes | Feedback on behavior | CT | Complete a quiz about physical activity levels and receive written feedback based on their score. | SB |
|  | OE 1.2. Understand the difference between physical activity and exercise | Framing/reframing | CT | Understand that exercise is only one of a range of physical activities that can promote health | S |
|  |  |  |  | Understand that exercise is only one of a range of physical activities that can promote health | S |
|  | KN 1.1. Recognize the benefits of PA | Information about health and emotional consequences | SCT | Know about the physical and emotional benefits of physical activity | SB |
|  |  |  |  | Know about the link between physical activity and sleep | SB |
|  | SE 1.1. Express confidence in being more physically active | Problem solving | SCT | Identify potential barriers to physical activity and generate strategies to overcome them | SB |
|  | SE 1.2. Express confidence in reducing sedentary behaviors | Instructions on how to perform the behavior | SCT | Recognize ways of reducing sedentary behaviors | B |
|  |  |  |  | Recognize ways of improving sleep quality | B |
|  | GO 1.1 Set a goal to reduce sedentary time | Behavioral goal setting | SCT | Set a SMART physical activity goal based on GO 4.1. | S |
| PO 2. Increase lifestyle activities | OE 2.1. Express confidence in (not) seeking social support | Problem solving and social support (unspecified) | SCT | Identify helpful and unhelpful social support towards PA changes (e.g. family, friends, health care professionals) | B |
|  |  |  |  | Problem solve potential social impediments | B |
| PO 2. Increase lifestyle activities (cont’d) | OE 2.2 Be aware that others also face difficulties being physically active after cancer treatment and increase motivation to be more active | Information about others’ approval | SCT | Read motivational quotes from other cancer survivors about physical activity | B |
|  | OE 2.3. Increase expectation that health care professionals will approve their lifestyle changes | Credible source | SCT | Read information from nurses in favor of physical activity | B |
|  | KN 2.1. Describe the PA recommendations | Instructions on how to perform the behavior | SCT | Know the PA recommendations | SB |
|  | SE 2.1. Express confidence in increasing lifestyle activities | Instructions on how to perform the behavior | SCT | Recognize ways of increasing lifestyle activities | SB |
|  | GO 2.1. Set a physical activity goal | Behavioral goal setting | SCT | Set a SMART physical activity goal based on GO 4.1. | S |
| PO 3. Increase organized activities | KN 3.1. Recognize safety issues while physically active | Instructions on how to perform the behavior | SCT | Recognize when and how to start and stop exercising | B |
|  | SE 3.1. Express confidence in doing more strength, balance, and flexibility exercises | Demonstration of the behavior & instructions on how to perform the behavior | SCT | Provide pictures of cancer survivors demonstrating sample strength, balance, and flexibility exercises and instructions of how to perform those | B |
|  |  | Behavioral practice/rehearsal | SCT | Practice some of the resistance exercises during the session following the booklet’s instructions | S |
| PO 3. Increase organized activities (cont’d) | GO 3.1. Plan in detail their organized PA | Problem solving and action planning | SCT | Recognize ways of organized PA | B |
|  |  |  |  | Identify what activities they could do | B |
|  |  |  |  | Examine where and when they will do the activity | B |
|  |  |  |  | Examine where and when they will do the activity | B |
|  |  |  |  | Examine if they need to involve others | B |
|  |  |  |  | Question if they will keep it up | B |
|  |  |  |  | Examine if they need to travel | B |
|  |  |  |  | Examine if it is safe to exercise | B |
|  | GO 3.2. Set a goal to increase organized activities | Behavioral goal setting | SCT | Set a SMART physical activity goal based on GO 4.1. | S |
| PO 4. Create and maintain a discrepancy-reducing feedback loop | OE 4.1. Express confidence in their ability to change their behavior | Pros and cons | SCT | Explain of the components of motivation to change | SB |
|  |  |  |  | Review examples of advantages and disadvantages for (not) changing their lifestyle | SB |
|  |  |  |  | Analyze their advantages and disadvantages of (not) changing their lifestyle | SB |
|  | OE. 4.2. Recognize the importance of making one behavior change at a time | Framing / reframing | CT | Recognize that making one behavior change at a time will be more advantageous in the long term compared to making too many changes at once | SB |
|  | OE 4.3. Recognize the value of self-monitoring and become aware of their PA pattern | Self-monitoring of behavior | CT | Verbal and written explanation that self-monitoring of behavior can improve behavior practice and maintenance | SB |
| PO 4. Create and maintain a discrepancy-reducing feedback loop (cont'd) |  |  |  | Be prompted to use a pedometer | SB |
|  |  |  |  | Provision of activity diaries | SB |
|  |  |  |  | Record the amount and type of moderate PA each day using (if preferred) the Shape-Up diary | SB |
|  |  |  |  | Record pedometer count | SB |
|  | OE 4.5. Express confidence in (not) seeking social support | Problem solving and social support (unspecified) | SCT | Identify helpful and unhelpful social support towards PA changes (e.g. family, friends, health care professionals) | B |
|  |  |  |  | Problem solve potential social impediments | B |
|  | OE 4.6. Plan their self-reward | Self-incentive & non-specific incentive | SCT | Understand the value of rewards for achieving their SMART goals | SB |
|  |  |  |  | Brainstorm ideas for non-food incentives | S |
|  |  |  |  | Plan to reward self when they review their goals if they achieve their SMART goals | SB |
|  | OE 4.7 Reward success | Social reward | SCT | Facilitator verbally praises participants if and only if participants have progressed with their goals | S |
|  | OE 4.8. Receive support from each other while making behavioral changes | Social support (unspecified) | SCT | Distribute to each other their contact details so that they support each other on the behavioral changes during the program | S |
|  |  |  |  | Be aware that others also face difficulties following a healthy lifestyle after cancer treatment | S |
|  |  |  |  | Get support and ideas from group members on following a healthy lifestyle | S |
| PO 4. Create and maintain a discrepancy-reducing feedback loop (cont'd) | OE 4.9. Identify potential sources of external support at the end of the program | Social support (unspecified) | SCT | Discuss if continuous support from other healthy lifestyle services can be helpful after the end of the program | S |
|  |  |  |  | Advice on how they can find information about support in their local area |  |
|  | SE 4.1. Express confidence in their ability to increase their willpower | Verbal and written persuasion about capabilities | SCT | Understand that willpower is a practicable skill (written)b | SB |
|  |  |  |  | Understand that they can learn to swap unhealthy habits to healthy onesa | S |
|  | SE 4.2. Express confidence in setting SMART healthy eating goals | Behavioral practice | SCT | Define the principles of SMART goal setting | SB |
|  |  |  |  | Practise translating vague goals to SMART ones | S |
|  | SE 4.3. Express confidence in rewarding themselves for achieving their SMART goals | Self-incentive & non-specific incentive | SCT | List their own potential rewards | B |
|  | SE 4.4. Decide if discrepancies exist when comparing PA to personal goals and guidelines | Discrepancy between current behavior and goal | CT | Compare their pedometer count and moderate PA (SE 5.2) to personal goals or guidelines in order to draw attention to the discrepancy. | B |
|  | SE 4.5 Maintain self-monitoring through diaries | Problem solving | SCT | Identify as a group barriers to keeping a food and activity diary | S |
|  |  |  |  | Discuss ways in which they can overcome the barriers | S |
| PO 4. Create and maintain a discrepancy-reducing feedback loop (cont'd) | SE 4.6. Express confidence in achieving SMART goals | Review of behavioral goals, problem solving, and social support (unspecified) | SCT | Re-examine their behaviors. Facilitator and group members provide evaluative feedback if they have (not) met the SMART criteria and if they have rewarded themselves for achieving the goal. Participants should problem-solve themselves with the help of the rest of the group ways of making their goals fit with the SMART criteria | S |
|  | GO 4.1. Compose an action plan towards increasing PA | Action planning and goal setting (behavior) | SCT | Plan in detail the performance of their first SMART goal | B |
|  |  |  |  | Plan things that might get in the way of achieving the goal | B |
|  |  |  |  | Plan how and when they will review the goal | B |
|  |  |  |  | Plan who might help them | B |
|  | GO 4.2. Compare PA to personal goals and guidelines | Self-monitoring on behavior and goal setting (behavior) | CT | Review their SMART goal | B |
|  |  |  |  | Evaluate outcome of their SMART goal | B |
| PO 5. Deal effectively with triggers of sedentary behaviors and lapses | SE 5.1. Express confidence in dealing with external triggers | Avoidance/reducing exposure to cues for the behavior | CT | Be prompted to identify and try to avoid cues promoting PA (e.g. watching a boxset) | SB |
|  |  | Distraction | CT | Be prompted to use an alternative focus for attention to avoid triggers for screen time | SB |
|  |  | Problem solving | SCT | Be prompted to identify the triggers for sedentary behaviors and generate a strategy to overcome them (e.g. have an alternative PA plan in case of foul weather) | SB |
|  | SE 5.2. Express confidence in dealing with fatigue | Problem solving | SCT | Understand what fatigue is | SB |
|  |  |  |  | Select strategies from a list of suggestions to help them overcome fatigue and facilitate physical activity | SB |
| PO 5. Deal effectively with triggers of sedentary behaviors and lapses (cont’d) | SE 5.3. Express confidence in dealing with unhelpful thoughts | Behavioral practice | SCT | Practice translating unhelpful thoughts that might impede healthy eating goals to helpful ones | SB |
|  |  | Self-talk | SCT | When experiencing an unhelpful thought, be prompted to remind themselves what they are trying to achieve and why | SB |
|  | SE 5.4. Express confidence in dealing with emotions | Distraction | CT | Identify ways for distracting from the mood | SB |
|  |  |  |  | Be prompted to focus attention on this alternative way when in the mood | SB |
|  |  | Behavioral substitution | CT | Be prompted to substitute screen time with other neutral or positive behaviors | SB |
|  |  | Reduce negative emotions | SCT | Be prompted to ask themselves whether not being active will change the problem/emotion they currently facing | SB |
|  | SE 5.5. Express confidence in dealing with lapses | Reduce negative emotions | SCT | Distinguish a behavioral lapse from a relapse | SB |
|  |  |  |  | Receive advice on the use of a 5-step cognitive strategy to reduce negative emotions in order to deal successfully with a lapse. | SB |
|  |  | Information about antecedents | SCT | Put a chain of antecedents (events, emotions, cognitions), lapse (sedentary behavior), and consequences in the right order. | S |
|  |  | Problem solving | SCT | Analyze the above factors, lapse, and consequences, and generate strategies to overcome them | S |
| PO 5 (cont’d) |  | Verbal persuasion about capability | SCT | Facilitator to reaffirm that participants can successfully overcome future lapses by reflecting on previous ones | S |
|  | GO 5.1. See GO 4.1 |  |  |  |  |
| Determinant-specific change objectives target each performance objective (PO). The determinants were outcome expectations (OE), knowledge (KN), self-efficacy (SE), and goals (GO). The BCTs and practical applications are present only in the sessions (S); both in the sessions and the booklet (SB); or only in the booklet (B). BCTs: Behavior Change Techniques, CT: Control Theory, SCT: Social Cognitive Theory. | | | | | |

Table S4 Structure and content of the “Shape-up following cancer treatment” sessions

| **# Session** | **Session title** | **Session content** | **Approx. time** |
| --- | --- | --- | --- |
| Session 1 | Preparing to Shape-Up | Welcome & introduction to the programme | 25min |
|  |  | Setting ground rules for the group | 10min |
|  |  | Discussion about previous experience of diet and physical activity changes, how cancer has shaped their eating and activity patterns, and hopes and fears about the programme | 15min |
|  |  | Motivation for change | 10min |
|  |  | Break | 5min |
|  |  | Setting a lifestyle target | 10min |
|  |  | Information and discussion about the importance of self-monitoring and food diaries | 10min |
|  |  | Round-up, and preparation for next session | 5min |
|  |  | Take home message | 5min |
| Session 2 | Keeping to a regular eating pattern | Review: Discussion about self-monitoring and food diaries, and goal progress | 20min |
|  |  | Volunteer-led discussion: Keeping to a regular eating pattern  Key learning points: The importance of keeping to a regular eating pattern, the definition of a regular eating pattern, the importance of breakfast, suggestions for goals, disadvantages of eating regularly. | 40min |
|  |  | Break | 5min |
|  |  | New topic: Goals and rewards  Discussion about the principles of goal-setting, group exercise about setting SMART goals, exercise about goal planning, discussion about rewards, and group exercise about non-food rewards | 15min |
|  |  | Round-up, and preparation for next session | 5min |
|  |  | Take home message | 5min |
| Session 3  Session 3 (cont’d) | Physical activity | Review: Discussion about keeping a diary, setting a regular eating goal, goal-setting, and rewards, goal progress | 20min |
|  |  | Volunteer-led discussion: Physical activity | 30min |
|  |  | Key learning points: The importance of physical activity for health and wellbeing, the difference between physical activity and exercise, goals to aim for (30 minute of moderate physical activity per day and muscle fitness exercises twice a week), the importance of incremental increase in physical activity levels. |  |
|  |  | Break | 10min |
|  |  | Setting an activity goal: individual exercise to help them focus on what they need to think about in order to improve their activity levels and group exercise for improving goal-setting skills | 15min |
|  |  | Round-up, and preparation for next session | 10min |
|  |  | Take home message | 5min |
| Session 4 | Eating a balanced diet | Review: Discussion about last week’s topics and creating a physical activity goal, goal progress | 20min |
|  |  | Volunteer-led discussion: Getting a healthier balance of foods.  Key learning points: the five food groups, what foods to make choose (Plenty of whole grains, fruits, and vegetables; moderate amounts from the “meat, fish and alternatives” and “milk and dairy” groups, preferably low-fat; have little amounts of “foods high in fat or sugar” and prefer healthy oils; limit processed meat, and sugary and alcoholic drinks; prefer foods low in salt). | 30min |
|  |  | Break | 5min |
|  |  | New topic: Lapses  Information, group exercise, and discussion on how to deal with lapses | 25min |
|  |  | Round-up, and preparation for next session | 5min |
|  |  | Take home message | 5min |
| Session 5 | Keeping an eye on food serving sizes | Review: Discussion about last week’s topics, goal progress, and exercise about managing lapses | 20min |
|  |  | Volunteer-led discussion: Keeping an eye on food serving sizes  Key learning points: Each participant brings a weighted portion of some foods and group members discuss about the understanding food serving sizes and how many servings they should aim for (The food servings are reflecting a 2,000kcal diet for women). | 50min |
|  |  | Break | 10min |
|  |  | Round-up, and preparation for next session | 5min |
|  |  | Take home message | 5min |
| Session 6 | External triggers | Review: Discussion about last week’s topics, and goal progress | 20min |
|  |  | Volunteer-led discussion: External triggers  Key learning points: The difference between external and internal triggers, and main strategies for dealing with external triggers | 35min |
|  |  | Break | 5min |
|  |  | New topic: Internal triggers  Discussion about hunger and cravings, group exercise for the dealing with cravings and the difference between craving and hunger, and discussion about fatigue | 20min |
|  |  | Round-up, and preparation for next session | 5min |
|  |  | Take home message | 5min |
| Session 7 | Internal triggers | Review: Discussion about last week’s topics, goal progress, and feelings about the group coming to an end | 20min |
|  |  | Volunteer-led discussion: Internal triggers  Key learning points: Definition of internal triggers (hunger, cravings, fatigue, emotions, and unhelpful thoughts) and strategies to deal with those | 40min |
|  |  | Break | 5min |
|  |  | New topic: Lapse chains  Information about lapse chains and group exercise of putting together a behaviour chain | 10min |
|  |  | Round-up, and preparation for next session | 10min |
|  |  | Take home message | 5min |
| Session 8  Session 8 (cont’d) | Food labels and the Shape-Up Change plan | Review: Discussion about last week’s topics, goal progress, and reviewing earlier areas of the programme | 10min |
|  |  | Volunteer-led discussion: Food labels  Key learning points: The ingredient list and the various names of sugar and saturated fat in food labels, the importance of checking the sugar, saturated fat, and salt content in the labels, and tips for smart shopping | 30min |
|  |  | Break | 5min |
|  |  | New topic: The Shape-Up Change plan  Information about how to complete a Shape-Up change plan and individual exercise on filling a mock plan | 20min |
|  |  | Discussion: Group members discuss their change plan, they re-evaluate their lifestyle target from Session 1, and are given information about the importance of continuing with self-monitoring | 20min |
|  |  | Round-up, and maintaining changes for the long term | 5min |
|  |  | Take home message | 5min |
| Reproduced with permission from [10]. | | | |

Table S5 Matrix for change objectives for effectively facilitating a group session (behavior)

| **Performance objectives** | **Change objectives** | **BCTs** | **Theory** | **Practical application** | **S/SM/M** |
| --- | --- | --- | --- | --- | --- |
| PO 1. Set up the group | OE 1.1. Understand the purpose of the program | Framing/reframing | CT | Written and verbal explanation of the scope and aims of the program | SM |
|  |  |  |  | Written and verbal explanation of the evidence-base behind the program | SM |
|  | KN 1.1. Understand what is needed to set up a group | Instructions on how to perform the behavior | SCT | Provision with detailed instructions on how to set up a group | SM |
|  | SE 1.1. Express confidence in setting up the group | Instructions on how to perform the behavior | SCT | Provision with detailed verbal and written instructions on how to set up a group | SM |
|  |  |  |  | Written and verbal suggestions for problem solving common issues | SM |
| PO 2. Run the group | OE 2.1. Understand the value of following the structured program intact | Framing/reframing | CT | Written and verbal explanation that the participants will benefit most if the program is being followed intact | SM |
|  | OE 2.2. Understand the responsibilities, perceived benefits as implementer | Framing/reframing | CT | Written explanation about their responsibilities as an implementer | M |
|  |  |  |  | Written explanation about potential benefits as an implementer | M |
|  | OE 2.3. Understand the value of the group format | Framing/reframing | CT | Written and verbal explanation about value of the group setting in promoting behavior change | SM |
|  |  |  |  | Written and verbal explanation about value of the self-help in a group setting | SM |
|  |  |  |  |  |  |
| PO 2. Run the group  (cont’d) | KN 2.1. Understand the program structure and components | Instructions on how to perform the behavior | SCT | Principles of facilitation | SM |
|  |  |  |  | Define the principles of SMART goal setting, self-monitoring, and self-incentives | SM |
|  |  |  |  | Provision with a detailed manual with detailed instructions of what to do in each session | SM |
|  | SE 2.1. Express confidence on running the program effectively | Instructions on how to perform the behavior | SCT | Provision with a detailed manual with detailed instructions of what to do in each session | SM |
|  |  |  |  | Written recommendations on dealing with issues / problems common to the “Shape-Up” groups | SM |
|  |  |  |  | Written recommendations on dealing with issues / problems common in any group setting | SM |
|  |  | Social support (unspecified) | SCT | Discuss in the facilitators’ forum arisen issues and problem solve potential solutions | SM |
|  |  |  |  | Contact program developers for arisen issues and problems | SM |
|  |  | Behavioral practice | SCT | Define the principles of SMART goal setting, self-monitoring, and self-incentives | SM |
|  |  |  |  | Practise translating vague goals to SMART ones | SM |
|  |  |  |  | Brainstorm potential self-incentives | S |
|  |  | Behavioral practice and problem solving | SCT | Practise problem solving common challenges in “Shape-Up” groups during the training session to increase skill | S |
| Determinant-specific change objectives target each performance objective (PO). The determinants were outcome expectations (OE), knowledge (KN), self-efficacy (SE), and goals (GO). The BCTs and practical applications are present only in the sessions (S); both in the sessions and the manual (SM); or only in the manual (M). BCTs: Behavior Change Techniques, CT: Control Theory, SCT: Social Cognitive Theory. | | | | | |

Table S6 Interview guide about the “Shape-Up” program (Adapted from [8])

| 1. How did you find the focus group discussion? (Only to focus group participants) |
| --- |
| 2. What one thing do you like the best? |
| 3. What one thing do you like the least? |
| 4. If you could change one thing about the materials, what would it be? |
| 5. What would get you to participate in the program? |
| 6. Suppose that you were trying to encourage a friend to participate in this program. What would you say? |
| 7. Within the adapted booklet, advice will be added specifically for endometrial cancer survivors. What are your views on: |
| a) Tailoring the information to endometrial cancer survivors = |
| b) Guidelines on managing GI symptoms (e.g. nausea, vomiting, diarrhoea) |
| c) Adding cooking classes to the program (to improve cooking skills) |
| d) Q&As re misconceptions and myths for nutrition and cancer (e.g. antioxidant supplements reduce recurrence) |
| 8. Who would you like to facilitate the group meetings? |
| 9. Would you prefer it as a group program or as a self-help? |
| 10. Do you have any other advice for us as we introduce this new program? |

**References**

1. Rock CL, Doyle C, Demark-Wahnefried W, Meyerhardt J, Courneya KS, Schwartz AL, Bandera EV, Hamilton KK, Grant B, McCullough M *et al*: **Nutrition and physical activity guidelines for cancer survivors**. *CA Cancer J Clin* 2012, **62**(4):243-274.

2. WCRF: **Food, Nutrition, Physical Activity and the Prevention of Cancer: A Global Perspective**. In*.* Washington, DC: World Cancer Research Fund / American Institute for Cancer Research; 2007.

3. Schmitz KH, Courneya KS, Matthews C, Demark-Wahnefried W, Galvao DA, Pinto BM, Irwin ML, Wolin KY, Segal RJ, Lucia A *et al*: **American College of Sports Medicine roundtable on exercise guidelines for cancer survivors**. *Med Sci Sports Exerc* 2010, **42**(7):1409-1426.

4. Koutoukidis DA, Beeken RJ, Lopes S, Knobf MT, Lanceley A: **Attitudes, challenges, and needs about diet and physical activity in endometrial cancer survivors: a qualitative study**. *Eur J Cancer Care (Engl)* 2016.

5. Braun V, Clarke V: **Using thematic analysis in psychology**. *Qualit Res Psychol* 2006, **3**:77-101.

6. Michie S, Abraham C, Whittington C, McAteer J, Gupta S: **Effective techniques in healthy eating and physical activity interventions: a meta-regression**. *Health Psychol* 2009, **28**(6):690-701.

7. French D, Olander E, Williams S, Fletcher H, Atkinson L, Turner A: **Building an evidence base for skills development training for cancer clinicians to support lifestyle behaviour change and self-management with cancer survivors**. In*.* Edited by Initiative NCS. Coventry, UK: Applied Research Centre in Health & Lifestyle Interventions, Faculty of Health & Life Sciences, Coventry University; 2011.

8. **Treating adult obesity through lifestyle change interventions: A briefing paper for commissioners** [<http://www.noo.org.uk/uploads/doc/vid_5189_Adult_weight_management_Final_220210.pdf>]

9. NICE: **Managing overweight and obesity in adults – lifestyle weight management services**. In*.* London: National Institute for Health and Care Excellence; 2014.

10. Koutoukidis DA, Beeken RJ, Manchanda R, Burnell M, Knobf MT, Lanceley A: **Diet and exercise in uterine cancer survivors (DEUS pilot) - piloting a healthy eating and physical activity program: study protocol for a randomized controlled trial**. *Trials* 2016, **17**(1):130.
